# High-grade bursal-side partial rotator cuff tears: comparison of mid- and long-term results following arthroscopic repair after conversion to a full-thickness tear

**DOI:** 10.1186/s13018-017-0619-7

**Published:** 2017-07-21

**Authors:** Nuri Aydin, Bedri Karaismailoglu

**Affiliations:** Orthopaedics and Traumatology Department, Istanbul University Cerrahpasa Medical Faculty, Kocamustafapasa Cad. No:53, Fatih, Istanbul, Turkey

**Keywords:** Partial rotator cuff tears, Bursal side, Shoulder arthroscopy, Arthroscopic repair, Surgical outcome

## Abstract

**Background:**

Partial-thickness rotator cuff tears (PTRCTs) are one of the leading causes of shoulder dysfunction. Successful results have been reported with different treatment techniques, but the long-term consequences of these procedures are not yet clearly known. The purposes of this study were to evaluate and compare the mid- and long-term clinical outcomes of arthroscopically repaired bursal-side PTRCTs after conversion to full-thickness tears and identify the possible effects of age, gender, and hand dominance on clinical outcomes.

**Methods:**

Twenty-nine patients who had undergone arthroscopic repair of a significant bursal-side PTRCT were functionally evaluated. The repair was made after conversion to a full-thickness tear. The average patient age was 55.2 years (range 35–69 years, SD ±7.6 years). Clinical outcomes were evaluated at 2 and 5 years after surgery. Constant Shoulder Score (CSS) and Visual Analogue Scale for Pain (VAS pain) were used as outcome measures.

**Results:**

The average CSS improved from 38.9 preoperatively to 89.2 and 87.8 at 2 and 5 years after surgery, respectively (*p* < 0.001). The average VAS pain score decreased from 7.90 preoperatively to 1.17 and 1.31 at 2 and 5 years after surgery, respectively (*p* < 0.001). A significant improvement was detected in patient functional outcomes and VAS pain scores at 2 and 5 years after surgery compared with the preoperative period. The patients who underwent surgery from their non-dominant extremity showed a significantly higher CSS increase relative to those who underwent surgery on the dominant extremity (*p* = 0.022).

**Conclusions:**

Arthroscopic repair of high-grade bursal-side PTRCTs after conversion to full-thickness tears is a reliable surgical technique with good functional outcomes and pain relief both at mid- and long-term follow-ups. Surgery on the non-dominant side may be related to better functional outcomes.

## Background

Arthroscopic repair of full-thickness rotator cuff tears has been reported to improve functional scores and promote healing [1–3]. Partial-thickness rotator cuff tears (PTRCTs) are characterized by a partial disruption in the tendon fibres. Ellman [4] classified PTRCTs arthroscopically according to the location (articular, bursal, or interstitial) and depth of the tear. Ellman grade III tears, which involve more than 6 mm or 50% of the tendon thickness, are also known as high-grade PTRCTs. Bursal-side tears typically occur in middle/older-aged patients (>40 years of age) as a result of intraarticular pathology or impingement and are less common than articular-side tears [5].

PTRCT management usually starts with conservative treatment, particularly when concomitant tendon and bursal inflammation are present. If the tear is deep and the symptoms are related to the tear rather than the inflammation, conservative treatment is rarely helpful, and early surgery is recommended by some authors [6]. Even if some factors such as age, activity level, tendon quality, and surgeon experience may affect the decision, debridement is generally preferred for Ellman grade I–II tears, whereas repair is indicated for high-grade (>50% depth) PTRCTs due to the limited healing capacity of the rotator cuff and the risk of tear progression [7]. Arthroscopic PTRCT repair is accomplished using the transtendinous technique or repair after conversion to a full-thickness tear [7, 8].

The main objective of this study was to evaluate and compare the mid- and long-term clinical outcomes of patients who underwent arthroscopic repair of bursal-side PTRCTs after conversion to full-thickness tears. The effects of patient age, gender, and hand dominance on the clinical outcome were investigated.

## Methods

After obtaining approval from the local Ethics Committee (06-01-2015/A-38), we retrospectively reviewed 42 patients (42 consecutive shoulders) who had bursal-side PTRCTs and underwent surgery between May 2009 and January 2012. All tears were treated arthroscopically using a single-row repair technique after conversion to a full-thickness tear.

### Patient selection

The inclusion criteria of this study were as follows: (1) pain and disability during daily living activities for at least 6 months, (2) no benefit from shoulder physiotherapy, (3) bursal-side partial supraspinatus tear evident on magnetic resonance imaging (MRI), and (4) high-grade (>50%) bursal-side partial supraspinatus tear diagnosed by intraoperative examination. The exclusion criteria were as follows: (1) previous surgery of the affected shoulder or cervical spine, (2) concomitant shoulder instability, (3) concomitant rheumatologic disease, or (4) concurrent shoulder procedures (repair of subscapularis, infraspinatus or teres minor tears, biceps tenotomy or tenodesis, distal clavicular resection, or labral repair). Twenty-nine patients met the inclusion criteria for the study and returned for postoperative clinical evaluations. Nine patients were lost to follow-up, and four patients did not meet the inclusion and exclusion criteria.

### Clinical evaluation

The patients were evaluated preoperatively and postoperatively at 2 and 5 years after surgery using the Constant Shoulder Score (CSS) and Visual Analogue Scale for Pain (VAS pain) and [9]. All of the pre- and postoperative evaluations were performed by the same surgeon. The data were collected prospectively during routine patient follow-ups, and the functional outcomes were investigated retrospectively according to patient age, gender, and hand dominance.

### Preoperative imaging

Preoperative radiographs of the shoulder were obtained in all patients to determine if any bony lesions or arthritic pathology were present. All patients were preoperatively evaluated by MRI to identify PTRCTs and diagnose concomitant abnormalities. The grade of rotator cuff tear was determined by intraoperative examination, and patients with tears larger than half (>50%) of the cuff thickness were included in this study [10].

### Surgical technique

The operations were performed under general anaesthesia with intravenous cefuroxime antibiotic prophylaxis in beach chair position by the first author. After inserting the arthroscope through a standard posterior portal, an anterior portal was established lateral to the coracoid process with the help of a spinal needle. This portal was used to assess any intraarticular pathologies. The lateral portal was established inferior to the lateral border of the acromion. After assessment of the glenohumeral joint, the arthroscope was redirected to the subacromial space. Subacromial decompression was performed using a radiofrequency device (Dyonics RF-S Whirlwind 90 Prob, Smith & Nephew, Memphis, USA). Acromioplasty was performed starting anteriorly and progressing posterolaterally in all patients using a 4.5-mm burr (Smith & Nephew, Memphis, USA) from the lateral portal. In the case of a curved or hooked acromion, a flat acromion was created. Degenerative tissue was debrided. The bursal-side PTRCT, which was determined preoperatively by MRI, was confirmed with intraoperative examination, and the grade of the tear was measured using a 6-mm arthroscopic probe. Bursal-side PTRCTs were converted to full-thickness tears with a 4.5-mm shaver. After preparing the footprint of the supraspinatus tendon over the bony tuberosity, an absorbable anchor (Twinfix AB 5.0 mm, Smith & Nephew, Memphis, USA) was placed at a 45° angle. The single-row suture anchor fixation technique was used in all tears. The sutures were passed through the tendons in a horizontal mattress fashion using arthroscopic suture passers (Elite Pass Suture Shuttle, Smith & Nephew, Memphis, USA) and tied with a sliding knot. The operations were completed at the closure of the portals with absorbable sutures.

### Postoperative rehabilitation

The patients were discharged on the same day or the day after the surgery. A standard postoperative rehabilitation protocol for full-thickness rotator cuff repair was followed. The arm was maintained in a simple arm sling for 6 weeks. Pendulum and self-assisted circumduction exercises were started immediately after the surgery. Passive range of motion exercises including abduction, external rotation, and forward elevation were initiated during the second postoperative week. Self-assisted active exercises were started at 6 weeks after surgery. Six months after surgery, patients were allowed to engage in heavy manual work and over-head activities.

### Statistical analyses

A paired *t* test was used to assess differences in pre- and postoperative CSSs. The analyses of variance (ANOVA) test was used to compare the results according to age, gender, and hand dominance. Statistical analyses were performed using SPSS program, version 22.0 (SPSS Inc.). Standard deviation (SD) was used as a measure of variability. The results are presented with two decimal places.

## Results

**Fig. 1:**
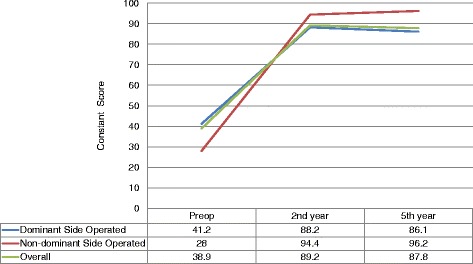
Graph comparing the mean CSSs calculated preoperatively and at 2 and 5 years after surgery and the difference regarding hand dominance

The mean patient age was 55.2 years (range 35–69 years, SD ±7.6 years), and the patient cohort included 20 females and 9 males. Five patients underwent surgery on their non-dominant sides, whereas the others underwent surgery on their dominant sides. No intra- or perioperative complications occurred, and none of the patients underwent revision surgery. Before surgery, the average CSS was 38.9 (range 20–68, SD ±11.6), and the average VAS pain score was 7.90 (range 6–10, SD ±0.81). The patients were re-evaluated at 2 and 5 years after surgery. At 2 years after the surgery, the average CSS was 89.2 (range 61–100, SD ±12.2), and the average VAS pain score was 1.17 (range 0–3, SD ±0.92). At 5 years after surgery, the average CSS was 87.8 (range 65–100, SD ±12.38), and the average VAS pain score was 1.31 (range 0–4, SD ±0.96). The CSS increases between the preoperative period and the 2nd postoperative year and between the preoperative period and the 5th postoperative year were both statistically significant (*p* < 0.001) (Table 1) (Fig. 1). Between the 2nd and 5th postoperative years, a slight decrease (1.4 points) in average CSSs was observed, but this change was not statistically significant (Fig. 1). The VAS pain score decrease was also statistically significant between the preoperative period and the 2nd postoperative year and between the preoperative period and the 5th postoperative year (both *p* < 0.001) (Table 1). The slight increase in average VAS pain score (0.14 points) between the 2nd and 5th postoperative years was not statistically significant.

**Table 1:** Significance of CSS and VAS pain score change over time by paired *t* test

	Mean difference	SD	*p* value
Constant Shoulder Score			
Preop–Postop 2nd year	−50.34	17.65	<0.001*
Preop–Postop 5th year	−48.93	19.29	<0.001*
Postop 2nd year–5th year	1.41	17.36	0.66
VAS Pain Score			
Preop–Postop 2nd year	6.72	1.16	<0.001*
Preop–Postop 5th year	6.58	1.18	<0.001*
Postop 2nd year–5th year	−0.13	0.99	0.46

CSS and VAS pain score improvements were statistically significant between the preoperative period and the 2nd postoperative year and between the preoperative period and the 5th postoperative year (both *p* **<** 0.001) *p < 0.05

*Preop* preoperative, *Postop* postoperative

When the hand dominance was considered, the patients who underwent surgery on their non-dominant sides showed significantly higher CSS increases between the preoperative period and the 2nd postoperative year (*p* = 0.022) and between the preoperative period and the 5th postoperative year (*p* = 0.017) compared with the patients who underwent surgery on their dominant sides (Table 2) (Fig. 1). Between the 2nd and 5th postoperative years, the patients who underwent surgery on their non-dominant sides showed a slight average CSS increase (1.80 points), whereas the patients who underwent surgery on their dominant sides showed a slight decrease (2.08 points), but these changes were not statistically significant (Fig. 1). Even though it was not statistically significant, the VAS pain score decrease in the patients who underwent surgery on their non-dominant sides continued between the 2nd and 5th postoperative years, whereas a slight increase was observed in the patients who underwent surgery on their dominant sides (Table 3).

**Table 2:** Average CSSs according to hand dominance, age, and gender (values; mean ± SD) with comparison of follow-up scores by ANOVA and the associated *p* values

Patient information		Average Constant Shoulder Score (±SD)					
		Preoperative	Postoperative		*p* value*		
			2 years	5 years	Pre–Po2	Pre–Po5	Po2–Po5
Operated extremity	Dominant side (*n* = 24)	41.16 (±2.17)	88.16 (±2.49)	86.08 (±2.49)	0.022*	0.017*	0.724
	Non-dominant side (*n* = 5)	28.0 (±4.75)	94.40 (±5.46)	96.20 (±5.46)			
Age	>55 years (*n* = 17)	36.70 (±9.99)	91.35 (±9.73)	91.33 (±2.04)	0.137	0.042*	0.673
	≤55 years (*n* = 12)	42.0 (±13.40)	86.25 (±15.05)	82.83 (±15.45)			
Gender	Female (*n* = 20)	38.60 (±10.15)	87.05 (±11.67)	89.00 (±11.77)	0.298	0.183	0.356
	Male (*n* = 9)	39.55 (±15.02)	94.11 (±12.71)	85.22 (±14.67)			

Patients who underwent surgery on their non-dominant extremities showed statistically significant CSS improvements between the preoperative period and the 2nd postoperative year and between the preoperative period and the 5th postoperative year (*p* < 0.05). Older patients (>55 years of age) showed greater CSS increases between the preoperative period and the 5th postoperative year relative to younger patients (*p* = 0.042) *p < 0.05

*Pre p*reoperative, *Po2* 2nd postoperative year, *Po5* 5th postoperative year

**Table 3:** Average VAS pain scores according to hand dominance, age, and gender (values; mean ± SD) with comparison of scores according to follow-up years by ANOVA and the associated *p* values

Patient information		VAS pain score (±SD)					
		Preoperative	Postoperative		*p* value*		
			2 years	5 years	Pre–Po2	Pre–Po5	Po2–Po5
Operated extremity	Dominant side (*n* = 24)	8.04 (±0.75)	1.29 (±0.95)	1.50 (±0.93)	0.661	0.824	0.423
	Non-dominant side (*n* = 5)	7.20 (±0.83)	0.60 (±0.45)	0.40 (±0.63)			
Age	>55 (*n* = 17)	8.12 (±0.85)	1.29 (±0.84)	1.47 (±0.94)	0.541	0.341	0.609
	≤55 (*n* = 12)	7.58 (±0.66)	1.00 (±1.04)	1.08 (±0.99)			
Gender	Female (*n* = 20)	7.90 (±0.85)	1.15 (±0.93)	1.40 (±1.04)	0.580	0.605	0.973
	Male (*n* = 9)	7.89 (±0.78)	1.22 (±0.97)	1.11 (±0.78)			

The VAS pain score change differences between groups were not statistically significant *p < 0.05

*Pre p*reoperative, *Po2* 2nd postoperative year, *Po5* 5th postoperative year

When patient age was considered, the differences in CSSs between the preoperative period and the 2nd postoperative year were not statistically significant. However, older patients (>55 years of age) maintained their clinical outcomes through the 5th postoperative year, whereas younger patients (≤55 years of age) showed a slight clinical deterioration. As a result, older patients showed significantly greater improvements in CSSs between the preoperative period and the 5th postoperative year compared with younger patients (*p* = 0.042) (Table 2). Males showed greater CSS improvements compared with females between the preoperative period and the 2nd postoperative year, although this difference was not statistically significant. Males showed a decrease in average CSSs between the 2nd and 5th postoperative years, whereas females showed a slight increase. However, these differences were not statistically significant (Table 2). VAS pain score change differences between groups regarding age and gender were also not statistically significant (Table 3).

## Discussion

In this study, statistically significant improvements in function and pain relief were detected in patients with bursal-side PTRCTs repaired arthroscopically after conversion to full-thickness tears, even 5 years after surgery. The change in functional outcome between the mid- and long-term follow-ups was not statistically significant. Undergoing surgery on the non-dominant side was found to be a possible factor that would positively affect the clinical outcome, although no statistically significant differences were observed regarding age and gender.

PTRCT repair may be performed either as a transtendon repair [11] or after conversion to a full-thickness tear [12]. No consensus in the literature exists regarding the best treatment choice. Some authors claim that conversion to a full-thickness tear and debridement of the degenerative tissue creates an environment with better healing capacity, similar to that observed for acute tears [13]. Some other authors prefer transtendon repair to preserve the remaining healthy fibres and restore the original rotator cuff footprint. In several studies comparing the two techniques, no significant differences in the functional outcomes and pain relief have been detected [8, 14]. In a recent meta-analysis of clinical results from articular-side PTRCTs treated with these two techniques, the authors reported no difference between clinical outcomes, but they did determine a higher re-tear rate in the group treated after conversion to full-thickness tears [15]. However, no similar results or meta-analysis exist for bursal-side PTRCTs in the literature.

Most of the previous studies have investigated articular-side PTRCTs, and fewer studies have reported functional outcomes following bursal-side tears. Bursal- and articular-side PTRCTs differ in their mechanisms of injury. It is believed that bursal-side tears are more related to an abnormal acromion causing extrinsic pressure over the tendon, whereas articular-side tears are more associated with intrinsic degeneration of the tendon itself [16]. Thus, the healing capacities between these two conditions may differ, and the outcomes of articular-side tears may not correlate with those of bursal-side tears. Upon closer inspection of the studies involving bursal-side tears, a controversy remains regarding which surgical technique yields the best clinical outcome. Koh et al. and Xiao and Cui achieved good clinical outcomes at 2-year follow-ups in their studies that included 38 and 48 patients, respectively, with high-grade bursal-side PTRCTs repaired arthroscopically by transtendon repair [11, 17]. Donohue et al. reported similar good results with articular, intratendinous, and bursal high-grade partial tears repaired arthroscopically after conversion to full-thickness tears in a study including 20 patients in each group [18]. In several studies, the authors compared the clinical results of bursal- and articular-side PTRCTs. Kim et al. did not determine any differences in re-tear rates between these two injuries but found superior clinical results at 2 years of follow-up for high-grade bursal-side tears compared with articular tears, which were treated after conversion to full-thickness tears [19]. Considering our results, this study has concluded that arthroscopic repair of bursal-side PTRCTs after conversion to full-thickness tears yields good clinical outcomes at both the mid- and long-term follow-ups.

Aleem et al. reviewed 55 patients who were operated bilaterally for full-thickness rotator cuff tears. Even though the clinical outcomes were better in the patients who underwent surgery on their non-dominant sides, the difference was not statistically significant [20]. In our study, the patients who underwent surgery on their non-dominant extremities showed a greater and statistically significant average CSS increase, revealing that hand dominance is an important factor for clinical outcome. Our results may be related to low patient adherence to postoperative activity limitations in those who underwent surgery on their dominant extremities. Although it was not statistically significant, the continuing decrease in VAS pain scores between the 2nd and 5th postoperative years in patients who underwent surgery on their non-dominant extremities, in contrast to the slight increase in the patients who underwent surgery on their dominant extremities, also supports this finding.

In our study, the average CSS improvement was statistically higher in older patients compared with younger patients not at the 2nd year but at the 5th year of follow-up. This is in contrast to the current literature, which claims that older patients have more persistent tears and younger patients have better clinical results [21, 22]. Our finding of a greater average CSS at long-term follow-up in older patients might be related to activity restrictions due to ageing, which allows for better healing in the repaired tendon.

In our study, males and young patients showed a decrease in average CSS between the 2nd and 5th postoperative years, but this difference was not statistically significant. This situation might be explained by the overuse of the operated extremity by young and male patients to a greater extent than old and female patients during their occupational, daily, and recreational activities.

Even though we observed significant clinical improvement in high-grade bursal-side PTRCTs treated arthroscopically after conversion to full-thickness tears, our study also had several limitations. First, no postoperative MRI or ultrasonography was obtained to investigate tendon integrity and possible re-ruptures. Second, there was no control group of patients treated with a different surgical technique to which we can compare our results. Therefore, it is not possible to conclude that the technique we used is superior to any others. Finally, the number of patients was small, and nine patients were lost to follow-up, which negatively affected the strength of our study.

Despite these limitations, this study has several strengths. First, all surgeries were performed by the same shoulder surgeon with the same surgical technique in the same group of patients with no additional lesions or interventions, which reduced the possible variability in clinical outcomes due to the surgeon, repair technique, or concomitant pathologies. Second, prospective data collection makes the results of this study reliable. Finally, to our knowledge, our study is the first to report the long-term results of high-grade bursal-side PTRCTs repaired arthroscopically after conversion to full-thickness tears and compare long-term results with mid-term results. Additionally, the effect of the relationship between hand dominance and operated side on clinical outcomes of high-grade bursal-side PTRCTs has not been reported in previous studies.

## Conclusion

Arthroscopic repair of bursal-side PTRCTs after conversion to full-thickness tears showed good functional outcomes and pain relief in long-term follow-up. The patients who underwent surgery on their non-dominant sides achieved better clinical outcomes than did the patients who underwent surgery on their dominant sides, and the difference was statistically significant. This situation might be related to lower patient compliance with activity limitations and extremity protection in those who underwent surgery on their dominant sides.
